# Review on Mechanoresponsive Smart Windows: Structures and Driving Modes

**DOI:** 10.3390/ma16020779

**Published:** 2023-01-12

**Authors:** Bo Chen, Qi Feng, Weiwei Liu, Yang Liu, Lili Yang, Dengteng Ge

**Affiliations:** 1 China Construction Advanced Technology Research Institute, China Construction Third Engineering Bureau Group Co., Ltd., Wuhan 430075, China; 2 State Key Laboratory for Modification of Chemical Fibers and Polymer Materials, College of Materials Science and Engineering, Donghua University, Shanghai 201620, China; 3 Institute of Functional Materials, Donghua University, Shanghai 201620, China

**Keywords:** energy saving, smart window, mechanoresponsive, driving mode

## Abstract

The growing awareness about the global energy crisis and extreme weather from global warming drives the development of smart windows market. Compared to conventional electrochromic, photochromic, or thermochromic smart windows, mechanoresponsive smart windows present advantages of simple construction, low cost, and excellent stability. In this review, we summarize recent developments in mechanoresponsive smart windows with a focus on the structures and properties. We outline the categories and discuss the advantages and disadvantages. Especially, we also summarize six unconventional driving modes to generate mechanical strain, including pneumatic, optical, thermal, electric, magnetic, and humidity modes. Lastly, we provide practical recommendations in prospects for future development. This review aims to provide a useful reference for the design of novel mechanoresponsive smart windows and accelerate their practical applications.

## 1. Introduction

With the continuous influence of energy crisis, the energy crunch in the whole world is furtherly aggravated. Additionally, with the increasing extreme weather caused by global warming, the development of science and technology to slow down global warming is also becoming a trend. Therefore, energy saving has been advocated by various countries. It is reported that the usage of building energy accounts for 30–40% of total energy consumption in developed countries [1,2], excessing the amount for industry or transportation. Around 47% energy for building service is contributed by heating, ventilation, and air conditioning systems [3,4]. As the main way of indoor–outdoor heat exchange, energy consumption through windows accounts for a large proportion. In China, the installed windows are 400–600 million square meters each year, exceeding the sum of that in the United States and European countries. Therefore, the design of smart windows that can dynamically modulate light transmittance is considered as an efficient approach for energy saving [5,6,7]. In addition to building energy saving, this technology has also attracted extensive interest in automobiles, greenhouses, and sunglasses, etc.

The radiated heat by sunlight in the visible and near-infrared wavelength accounts for about 44% and 47% of total radiated heat, respectively. Upon external stimulus, smart windows can reversibly adjust the light reflectance or transmittance in visible or near-infrared wavelength [8]. Proposed by Lampert et al. in the 1980s [9], smart windows based on electrochromic materials is the most attractive type and have the widest applications. It typically presents a sandwich structure with a functional material layer between two transparent electrodes. The optical properties are tunable due to the characteristic of redox reaction or ion embedding/detachment under electric field [10]. The thermochromic materials can change the colors with the temperature due to the thermal decomposition or geometrical conformation change [11]. When exposed to specific wavelengths of light, photochromic materials can change color resulting from the chemical bond homolytic reaction or pericyclic reaction [12]. Despite extensive research in electrochromic, thermochromic, and photochromic smart windows, the fabrication and construction are rather complicated and require external fields to achieve the optical modulation, limiting the commercialization of these materials. In recent years, mechanoresponsive smart windows tuned by simple mechanical strain have attracted increasing interest. Usually, the surface morphologies or internal structures of mechanoresponsive materials are deformed or reconfigured due to the mechanical strain, leading to the change in light transmittance or color through light scattering or diffraction. Therefore, there is usually no external power supply required for control, allowing the cost-effectiveness for mass production. Moreover, the optical properties of mechanochromic materials are able to be tuned even at very small strain (i.e., ~10%). In contrast, electrochromic, photochromic, or thermochromic smart windows generally need more time for response. For example, electrochromic WO_3_ film exhibits coloration/bleaching time of 1~6 s. Thermochromic poly-(N-siopropylacrylmide) (PNIPAm)-based films demonstrate a response time of 40~120 s. Inorganic photochromic films show a wide range of response times, from 0.1 s to hundreds of seconds.

In this review, we summarized recent developments in mechanochromic smart windows. According to the surface/interface mechanism, we discuss the strategies for all six types of mechanochromic smart windows. The most common driving mode to mechanical strain is mechanical force, i.e., changing the surface/interface morphology by stretching, compressing, rotating, etc. Here, we also summarize six unconventional driving modes to generate mechanical strain and discuss the advantages and disadvantages. Lastly, the potential applications in the mechanoresponsive smart windows are provided for future development.

## 2. Structures of Mechanoresponsive Smart Windows

Mechanical strain driven by mechanical force, electricity, humidity, etc., reconfigures the surface morphology or changes the interface structure, thus changing the optical transmittance by modulating the scattering or diffraction of lights. Based on the types of surface morphology or interface structure to achieve optical modulation, we created six primary categories of mechanoresponsive materials: micro/nano-array, wrinkling, crack, novel interface, tunable interface parameter, and the surface–interface synergy effect. For each category, we focus on the latest developments in the mechanisms and optical tunable properties.

### 2.1. Mechanoresponsive Smart Windows Based on Micro/Nano-Array

Surfaces periodic nanoscale or microscale arrays have attracted attention as a new type of smart windows material, because of their special advantages, such as flexible light trapping property, broadband antireflection, easy preparation, and high surface area. To date, several nanoarrays have been designed and fabricated, such as nanocones [13], nanopillars [14,15,16], nanoholes [17,18], nanospheres [19]. These structures can change their optical performance by dynamically modulating surface geometry in response to various external stimuli.

**Figure 1:**
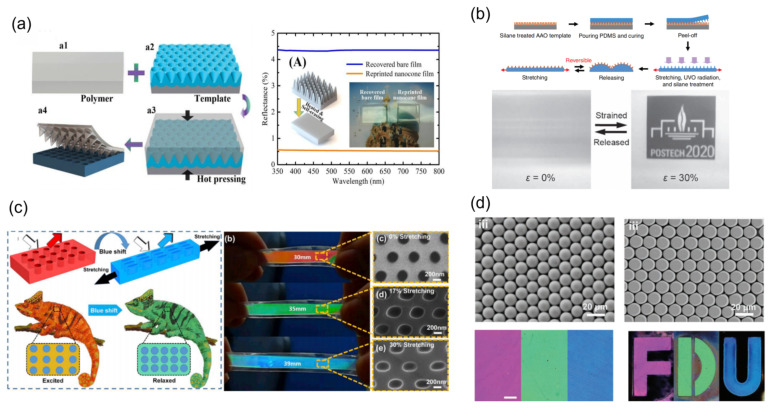
The strategies of mechanoresponsive smart windows based on nanoarrays: (**a**) nanocone [20] Copyright 2018, MDPI (Basel, Switzerland), (**b**) nanopillar [21] Copyright 2010, John Wiley and Sons (Hoboken, NJ, USA), (**c**) nanoholes [22] Copyright 2019, Elsevier (Amsterdam, The Netherlands), (**d**) nanospheres [23] Copyright 2021, John Wiley and Sons.

In recent years, Li et al. reported a self-erasable nanocone antireflection film via a simple surface replication method (Figure 1a) [20]. The nanocone arrays with constant heights and periods can be easily formed in the shape memory polymer film of polyvinyl alcohol (PVA) and are conveniently erased by thermal irritation. In the initial state, the PVA film with shape memory effect shows 0.6% reflectivity in the visible spectral, and the reflectivity of self-erasable PVA film can be switched from 0.6% to 4.5% by adjusting the temperature (>80 °C). Benefiting from PVA’s shape memory effect, the reflectivity over the visible spectral range of the self-erasable antireflection membrane can be changed. Lee et al. presented a simple manufacturing of stretchable smart windows with the surface morphology pattern consisting of nanopillar arrays on the wrinkled poly(dimethylsiloxane) (PDMS) film [21]. As shown in Figure 1b, in released state, because of the wide scattering of light via the periodic micrometer size surface structures, the PDMS films show optically opaque properties, which have a frosted glass-like appearance.

Under the 30% mechanical stretch, the films became optically transparent and the transmission was up to 95%, due to the absence of wrinkles. Inspired by chameleons, which can adjust their colors according to the environment, Zhao et al. designed a surface nanohole-type color display film [22]. As shown in Figure 1c, the stretchable nanohole photonic crystals are prepared by nanoimprint technology. The compound film with shape memory function is prepared via using shape memory alloy (SMA) and PDMS materials. The function of SMA is to make the nanohole photonic crystal deform under the electrical stimulation and the function of PDMS is to make the SMA restore its initial shape after taking off the electrical stimulation. Under the voltage of 1.0–1.5 V, the compound film can achieve color changing over the whole visible light range, and the total deformation required is only less than 30%. Ji et al. reported a nanospheres shape memory retro-reflective structural color film that originated from the mechanisms of retro-reflection and thin-film interference combined with the internal reflection (Figure 1d). During the deformation process, due to the change in nanospheres shape, the structural color of the optical film gradually disappears [23]. Moreover, this film displays excellent repeat and rewrite ability, which structural color can be recovered by heating. In summary, micro/nano-array-based mechanoresponsive smart windows can adjust color or transparency as the tunable array shape or array numbers simply. However, their optical modulation is limited, because conventional micro/nano arrays are difficult to disappear completely in the process of deformation.

### 2.2. Mechanoresponsive Smart Windows Based on Wrinkling

As a universal pattern in nature, wrinkling is another typical surface texture that can dynamically be tuned by mechanical strains and has been extensively explored for optical devices, stretchable electronics, energy storage devices, etc. [24,25,26]. Depending on the incident angles, the wrinkled surface refracts the incident light to different directions and blurs the objects behind the wrinkled thin layer/substrate film. However, when the wrinkles are erased by lateral tensile strains, the light beams can pass through the film with reduced deflection, changing the film from opaque to translucent or to highly transparent [27,28].

**Figure 2:**
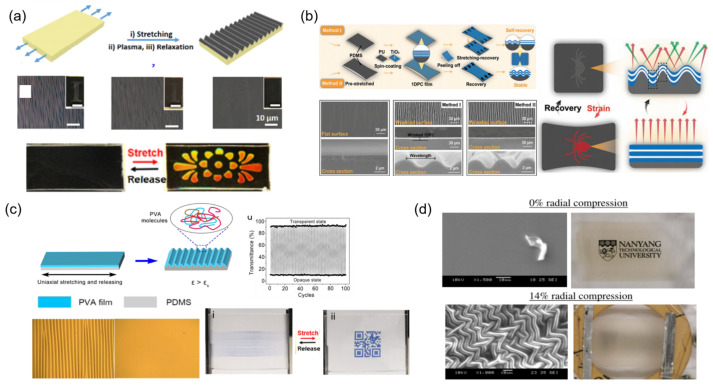
The strategies of mechanoresponsive smart windows based on wrinkling: (**a**) stretching to form wrinkles [29] Copyright 2020, Springer Nature (Berlin/Heidelberg, Germany), (**b**) wrinkled photonic elastomer structure [30] Copyright 2022, John Wiley and Sons, (**c**) double-layer film wrinkle [31] Copyright 2019, American Chemical Society (Washington, DC, USA) (**d**) biaxial compression to form wrinkles [32] Reprinted with permission Copyright 2016, Optical Society America (Washington, DC, USA).

In recent years, researchers have designed a variety of surface wrinkled smart window materials. In Figure 2a, Li et al. reported a notable thickness-dependent wrinkling behavior of PDMS films via using the typical plasma-stretch processes [29]. They have showed brilliant surface structural colors and pre-designed colorimetric responses to mechanical strain on plasma-treated PDMS films by changing the substrate thickness. Because of the high orderliness and considerable small size of the wrinkles, uniform, bright, and angle-dependent structural colors can be obtained on thick PDMS films (>1 mm).

To eliminate the angle dependence of the structural color, inspired by a kind of bright blue luminescence spider, Lin et al. reported a new wrinkle-based photonic elastomer structure [30]. Through wrinkling stretchable 1D photonic crystals (1D PC), the photonic elastomers film with omnidirectional angle-independent brilliant structural colors are achieved (Figure 2b). They used stretchable polyurethane and nanoscale TiO_2_ as the raw materials by the method of alternating assembly to fabricate 1D PC as the surface structural color layer, and PDMS as the bottom elastic layer which enables the discoloration response of elastomers to wrinkles with clear boundaries. The wrinkle-based structural color and photonic structure can remain stable after 1000 tensile cycles, and mechanochromic sensitivity up to 3.25 nm/%. More importantly, through comprehensively controlling the lattice spacing of photonic films and micro-wrinkle structure, the structural color can realize delayed discoloration and reversible switching performance only by a single strain direction. As shown in Figure 2c, Jing et al. reported an effective strategy to preparing temperature and moisture dual-responsive surface wrinkles based on the PVA/PDMS bilayer film, which can be achieved by the rational design of modulus changing PVA skin layer on elastomer film upon moisture and temperature [31]. The bilayer film systems show remarkable advances in a fast response system, outstanding reversibility, stability, and high light transmittance modulation. Compared to the examples of uniaxial strain to form wrinkles, the biaxial compression can generate more complex 2D surface wrinkling patterns, which could be ordered or disordered. The 2D wrinkling patterns have greater interactions with lights and result in a lower transmittance. In Figure 2d, Shrestha et al. displayed a flat ZnO thin film which can be deposited on a pre-stretched elastomer membrane acrylic elastomer membrane by the electron beam evaporation technique [32]. This optical tunable window film appears reversible and adjustable between translucent and transparent states. When compression is not applied to a flat surface, the film is transparent with a 93% transmittance at a wavelength of 550 nm. At 14% radial compression, the film appears to surface wrinkle and has a translucent appearance, which has a very low 3% in-line transmittance. Analysis shows that both the large amplitude and the small wavelength of transparent micro-wrinkles are in favor of refracting light diffusely.

### 2.3. Mechanoresponsive Smart Windows Based on Cracks

In addition to optical property modulation by changing the structural parameters such as the number and micro/nano-pattern spacing on the surface, optical pathway opening/closing or the appearance/disappearance of scattered light units by changing the opening/closing state of micro cracks is also an effective means to achieve rapid excitation of fluorescence or rapid decrease of transmittance [33]. Because of the weak binding at the fracture, it usually has highly sensitive properties. Both microscale and nanoscale structural designs have well demonstrated their opportunity in achieving excellent dynamic optical performance. By means of stretching/releasing and revealing/concealing patterns, micro/nanoscale cracks enable tune a series of reversible adaptive optics, e.g., transparency, fluorescent color, and luminescent intensity.

**Figure 3:**
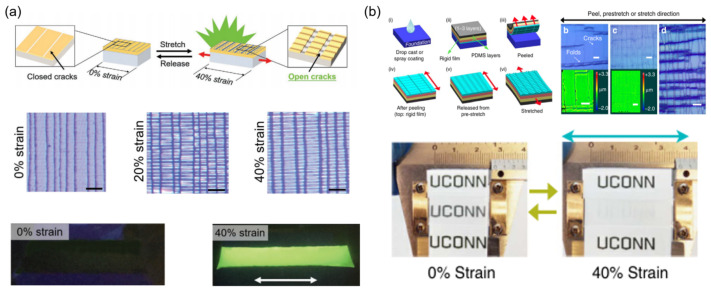
Construction paths of mechanoresponsive smart windows based on surface crack: (**a**) surface deposition of metal coating [34] Copyright 2017, John Wiley and Sons, (**b**) soft substrate/ hard shell [35] Copyright 2017, John Wiley and Sons.

Mao et al. reported a simple and highly effective method of mechanochromic materials based on a bilayer structure. This structure is composed of a sputter-coated light-shielding metal layer (Au/Pd) on a PDMS bottom substrate containing the fluorescent dye [34], featuring horizontal and/or vertical micro-scale cracks under stretched material (Figure 3a). The width of cracks opening on the metal light-shielding layer under stretching/releasing endows the UV radiation to stimulate the fluorescent dye, which can exhibit luminescent color embed in the PDMS matrix. The crack opening width can be well tuned via applying different degrees of pre-stretching strain in the preparation process, leading to customizable mechanochromic responses. High sensitivity and excellent durability of the devices are also displayed, which can exhibit great mechanochromic properties after 500 cycles of tensile and release. Zeng et al. introduced a thin rigid composite material film, which was prepared by drop-casting method or spray-coating on the plastic substrate subsequent to the treatment of vinyl-functionalized silane vapor [35]. The surface morphology of the rigid film showed periodical longitudinal cracks which are vertical to the peeling direction and the transverse wrinkles perpendicular to the cracks because of the compressive force originating from the Poisson effect. In the released state, this film shows >88% transmittance at 600 nm and the film becomes highly opaque (transmittance < 29%) under 40% strain (Figure 3b).

### 2.4. Novel Interface-Introduced Mechanoresponsive Smart Windows

Similar to the surface structure, the interface structure can also dynamically regulate the scattering and interference of light, thus achieving the optical modulation. Moreover, the mechanoresponsive materials based on interface structure regulation can overcome the problem that the surface structure is susceptible to failure by external environment (such as dust, moisture, mechanical load, etc.), and show stronger controllability and stability [36,37]. Based on this, materials that achieve light transmission regulation through dynamic generation/disappearance of novel interfaces have been widely investigated.

**Figure 4:**
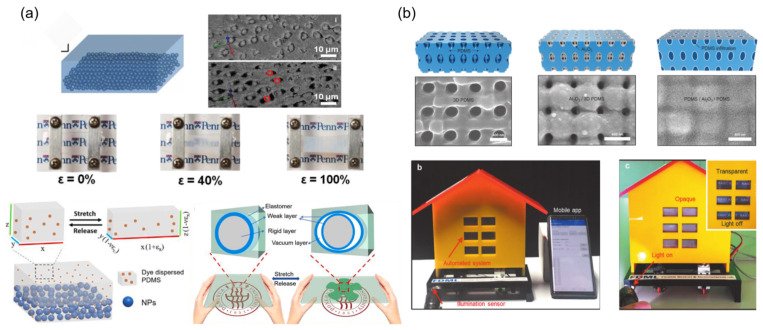
Methods to novel interface dynamic generation: (**a**,**b**) phase separation due to huge elastic modulus difference [38,39,40] Copyright 2015, John Wiley and Sons, 2021, Elsevier and 2020, John Wiley and Sons.

As shown in Figure 4a, Shu Yang’s group and our group firstly proposed the approach to prepare smart window film by combining the nanoparticle arrays with similar refractive indices to elastomers [38]. During the stretching process, a large number of micro/nano-optical interfaces were generated because of the mismatch between the modulus of the soft elastomer and the rigid nanoparticles, which result in a significant decrease in the light transmission of the film. Although the transmittance of this film is up to 70%, it still improves the mechano-optical sensitivity. Therefore, constructing fast-responsive interfaces through the generating/vanishment of scattering under mechanical strain, is an easy and effective way to improve sensitivity. Based on this theory, our group reported an ultrasensitive dynamic optical membrane based on the dye-induced weak boundary layer [39]. This sample exhibits a dramatic decrease in transmittance by 44% at very small strain (15%). Moreover, a total dynamic transmittance rate of ~75% is demonstrated, while this membrane can be reversibly modulated for more than 2000 cycles with stable structural integrity and optical performance.

Cho et al. reported a new type of 3D nanoscale composite film, consisting of an ultrathin Al_2_O_3_ nanoshell [40]. Regardless of the stretching direction, a large amount of light-scattering nanogaps form at the interfaces of Al_2_O_3_ and the elastomers under stretching (Figure 4b). These result in the dramatic modulation of transmission from a high 90% to a very low 16% at visible wavelengths and does not attenuate after the stretching/releasing of more than 10,000 cycles.

### 2.5. Mechanoresponsive Smart Windows Based on Tunable Interface Parameters

In addition to the generation/disappearance of novel interfaces, the regulation of interfacial structure parameters is also an important factor to change the diffraction and light transmission direction. Typically, there are three types of interface structure parameters: the interface spacing, the interface shape, and the alignment direction.

**Figure 5:**
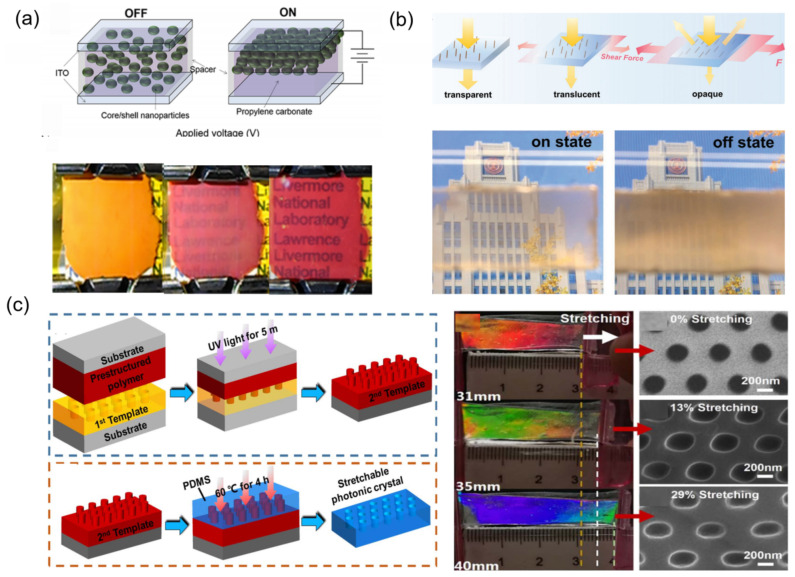
Mechanoresponsive smart windows with dynamic control of interface parameters: (**a**) colloidal particle spacing [41] Copyright 2018, American Chemical Society, (**b**) colloidal particle direction [42] Copyright 2021, John Wiley and Sons, (**c**) spacing and shape of hole [17] Copyright 2019, Iop Publishing (Bristol, UK).

In Figure 5a, Han et al. used colloidal spherical nanoparticles with core-shell structure to successfully synthesize a transparency tunable film in response to electric stimuli [41]. They demonstrated a suspended particles device, which can tune the transparency in the visible wavelength by using colloidal assemblies of nanoparticles. The change in observed transparency can be attributed to the tunable structural ordering of nanoparticle assemblies and the modulation of photonic band structures. Moreover, the macroscopic structure color was able to be changed through regulating the band gap center wavelength along with the lattice constant of nanostructures. As shown in Figure 5b, Li et al. reported a new highly sensitive, shear-responsive smart window, which consists of vertically fixing the Fe_3_O_4_@SiO_2_ nanochains’ (NCs) array and an elastic matrix of polyacrylamide. At original relaxation state, all Fe_3_O_4_@SiO_2_ nanochains stand vertically to the film surface and this flexible film shows optical transparency. When the strain is applied, Fe_3_O_4_@SiO_2_ nanochains tilt along the shearing direction, which enables a good shielding effect. Critically, a quite small shear displacement up to 1.5 mm applied on the surface will give rise to tunable optical states, changing from the high transparency state of 65% transmittance to the opaque state of 10% transmittance [42]. In Figure 5c, Zhao et al. designed and fabricated a stretchable photonic crystal via nanoimprinting technology. Periodic cylinder-shaped air holes were embedded in the non-close-packed triangular lattice. This film can switch color over the whole visible light range from red to blue color under a small, applied strain of 29%. In addition, a reversible stretching up to 2000 times also exhibits the stability of shape recovery as well as mechanochromic ability [17].

### 2.6. Mechanoresponsive Smart Windows Based on Surface–Interface Synergy Effect

In order to enhance the optical modulation range and achieve a multi-state, the synergistic surface–interface modulation is an effective approach. It is possible to achieve color and transmittance modulation based on multiple effects at the same time. Therefore, surface–interface synergy effect has unique advantages in multi-state display and precise regulation.

**Figure 6:**
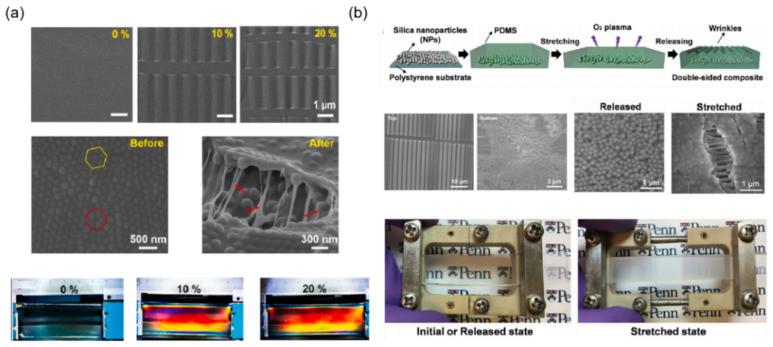
Two strategies of mechanoresponsive smart windows based on surface and interface modulation synergistically: (**a**) surface wrinkle, novel interface dynamic formation dynamic control of interface parameters [43] Copyright 2021, Elsevier, (**b**) surface wrinkle and novel interface dynamic generation [44] Copyright 2018, John Wiley and Sons.

As shown in Figure 6a, a large-area mechanochromic film is reported by Qi et al. based on a novel design of bilayer PDMS film including angle-independent and angle-dependent structural colors through bottom-up bar coating [43]. The angle-independent structural color is attributed to the long-range disordered but short-range ordered structure of polystyrene (PS) nanoarrays. Meanwhile, angle-dependent structural color is generated due to the stretching of surface wrinkling. Moreover, the cracks and surface wrinkles of PS nanoarrays resulting from the tensile enhance the scattering effect of bilayer film and reduce the transmittance of the light. Furthermore, pressure-induced surface morphology rearrangements can remove the wrinkling behavior. Therefore, the properties of programmable mechanochromic responses can be achieved. The pressure-encoded invisible complex information can be reversibly displayed by stretching.

In order to realize the highest transmittance modulation under the special application requirements, Kim et al. presented a novel strategy to prepare an on-demand smart window by integrating the synergetic optical effects due to the tunable wrinkled geometry and nanovoids generated by the surrounding silica particles embedded in PDMS film (Figure 6b) [44]. By carefully varying the wrinkle shape, the size of silica particle and stretching strain, a great optical transmittance modulation in the visible band to near infrared range is realized, while with a relatively small strain up to 10%. At 0% strain, the film shows 60.5% transmittance at the wavelength of 550 nm due to the light diffraction caused by the initial wrinkles. Upon stretching to the pre-strain level (10%), a maximum transmittance (86.4%) is obtained at a visible wavelength of 550 nm. While at 40% strain level, the film demonstrates a significantly low transmittance (25.2%).

## 3. Driving Modes of Mechanoresponsive Smart Windows

Due to the surface morphology or interface structure regulation via mechanical strain, the optical modulation is achieved. The common driving mode of mechanical strain is mechanical force, i.e., stretching, compressing, and rotation. However, this mode needs the physical contact under deformation. Here, we summarize six unconventional modes to drive mechanical strain: pneumatic, optical, thermal, electric, magnetic, and humidity. We focus on the strategies and the optical modulation properties.

### 3.1. Pneumatic Driving Mode

Pneumatic deformation devices are commonly found in soft robots, which include a sample holder, a vent, a manometer, and an air injection device. In this case, the sample is changed in a biaxial strain-like manner. As a result, a small amount of gas injection can lead to large three-dimensional directional deformations, thus enabling rapid tuning of optical properties.

**Figure 7:**
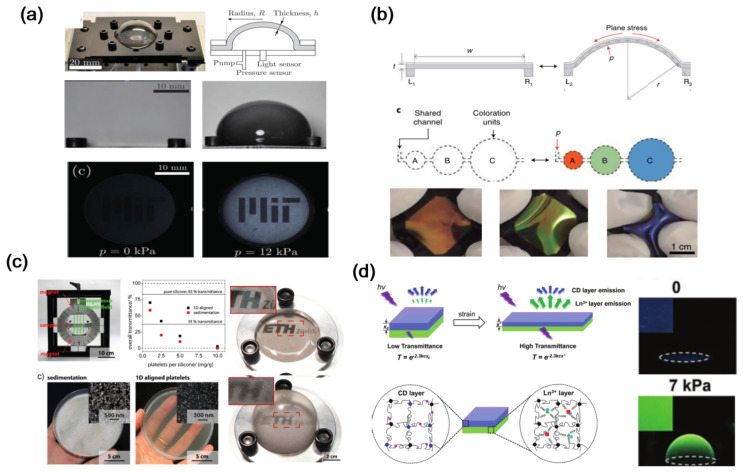
The strategy of pneumatic drive mode mechanoresponsive smart windows: (**a**,**b**) adjust transparency and color by changing film thickness [45,46] Copyright 2016, John Wiley and Sons and 2021, Springer Nature, (**c**) pneumatic stretching to adjust transparency [47] Copyright 2014, John Wiley and Sons, (**d**) pneumatic drive mode mechanochromic hydrogels [48] Copyright 2019, John Wiley and Sons.

As shown in Figure 7a [45], Jimenez et al. designed a class of soft color composite device in which the light transmittance can be dynamically tuned and regulated through mechanical actuation. In order to provide tunable opacity, it consists of thin sheets of PDMS embedded with colloidal suspension of black dye particles of micrometer size. The thickness of this device can be tuned pneumatically, and subsequently, the transmittance of this device can also be changed. Similarly, the thin films of chiral liquid crystalline elastomers also demonstrate the transmittance modulation via pneumatically inflating [46]. Benefiting from the large Poisson’ s ratio (>0.5) and elasticity anisotropy of these materials, the size or the layout of encapsulated air channels are geometrically programmed, leading to color change from near-infrared to ultraviolet wavelengths while the transverse strain is less than 20% (Figure 7b). In Figure 7c, Rotzetter et al. presented the polymer composites membrane including aluminum flakes, which can tune their light transmittance upon pneumatic stretching [47]. They added magnetic aluminum flakes into one kind of polymer matrix and controlled the direction of the platelets via applying an externally homogeneous magnetic field during the curing of polymer matrix. This endowed the irreversible fixation of magnetically oriented flakes in the polymer matrix layer. Therefore, the flakes’ direction can be further adjusted when the polymer matrix is expanded. Based on this mechanism, flexible silicone composites were fabricated, which displayed a decrease in light transmittance during pneumatic stretching. In Figure 7d, based on a novel double-layer luminescent spectra design, luminescent and mechanochromic hydrogels were reported by Zhu et al. [48]. In the hydrogel, carbon nanodots and Ln ions were also used as the luminescent species. In case of pneumatic driving, the thickness of the top layer is reduced by virtue of the Poisson’ s effect, enhancing the transmittance of the bottom layer in which luminescence in turn increases. Here the relative increase in transmittance depends on the primitive top-layer absorbance, the mechanochromism can be changed simply by adjusting the carbon nanodots concentration in the top layer. Pneumatic driving mode smart windows change light transmittance via applying or removing air pressure, greatly enhancing building energy conservation.

### 3.2. Optical Driving Mode

Optical driving represents a typical actuation method that is non-contact, easy to regulate locally, and can be regulated remotely. In addition to deformation control by light-induced molecular reactions, deformation of the substrate elastomer by photothermal effects is an effective means to achieve surface structure control.

**Figure 8:**
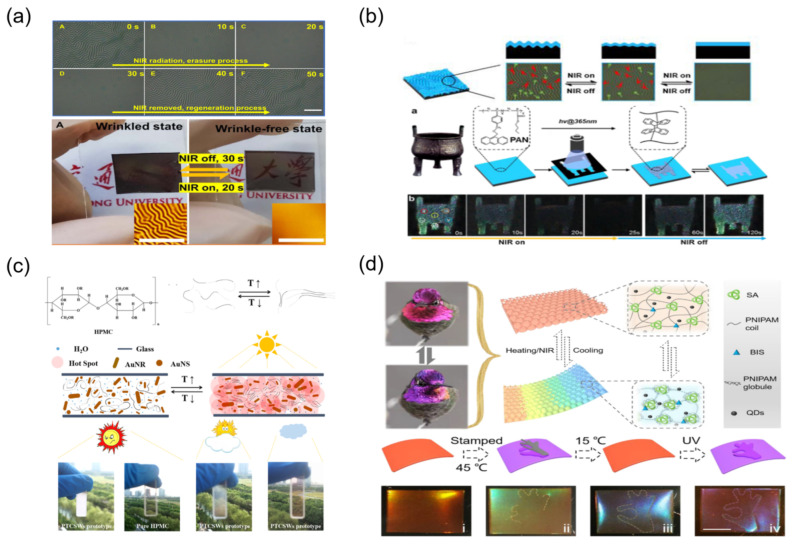
The approaches for optical driving mechanoresponsive smart windows: (**a**,**b**) based on the high photothermal conversion efficiency of CNT [49,50] Copyright 2018, American Association Advancement Science (Washington, DC, USA) and 2020, American Chemical Society, (**c**) based on metal nanoparticles [51] Copyright 2018, John Wiley and Sons, (**d**) based on inverse opal scaffold structure [52] Copright 2022, Elsevier.

Optical driving dynamic micro/nano patterns pave an effective strategy for the on-demand adjusting of surface properties to achieve a smart surface. In Figure 8a, Li et al. generated a surface dynamic wrinkling via near-infrared (NIR) light, based on the soft substrate of carbon nanotube (CNT) containing PDMS elastomer and various functional polymers as the top stiff layer of bilayer systems [49]. The excellent NIR photo-thermal effect of CNT results in a great thermal expansion of elastic CNT-PDMS substrate. Subsequently, the surface morphology structures can be reversibly tuned from the transparent state to the opaque state under NIR irradiation in a very short time. This facile but effective strategy via utilizing a reversible surface pattern from NIR-driven wrinkles can be used for security. As illustrated in Figure 8b, the hidden information can reversibly transform from on to off due to the dynamic nature of fingerprint-like wrinkles, and their NIR-responsiveness can maintain or remove the colorful patterns, thus truly realizing the security [50]. In Figure 8c, Cao et al. proposed a simple and economy method to achieve reversible switch of color or transparency, which integrate the noble metal nanoparticles’ photothermal conversion with thermochromic compounds [51]. Compared with the traditional laser irradiation, the sunlight irradiation is very environmentally friendly. In particular, they realized a photo-thermochromic smart windows prototype driven by environment sunlight, in which it can automatically change to opaque to block sunlight on burning days and return to the transparent state on cloudy or other low lighting conditions. As shown in Figure 4d, Wang et al. designed a shape-memory structural color film via introducing shape memory polymers of N-isopropylacrylamide and stearyl acrylate into inverse opal scaffold features [52]. With the support of a photothermal effect of graphene, this film displays reversible deformation and structural color upon the photo irradiation. The optical driving mode has the advantage of energy saving and convenience. However, their original color cannot be recovered only by the removing of UV, which would restrict their practical applications as smart windows.

### 3.3. Thermal Driving Mode

Heat is a widespread energy source in life, and traditional thermochromic materials often achieve color transformation through thermally induced changes in molecular structure and crystalline state. In contrast, thermally driven deformation is often based on shape memory polymers, whose function is mainly based on the existence of two phases within the material that are not fully compatible, i.e., a fixed phase that maintains the shape of the finished product and a reversible phase that softens/hardens and changes reversibly with temperature changes [53,54,55,56,57].

**Figure 9:**
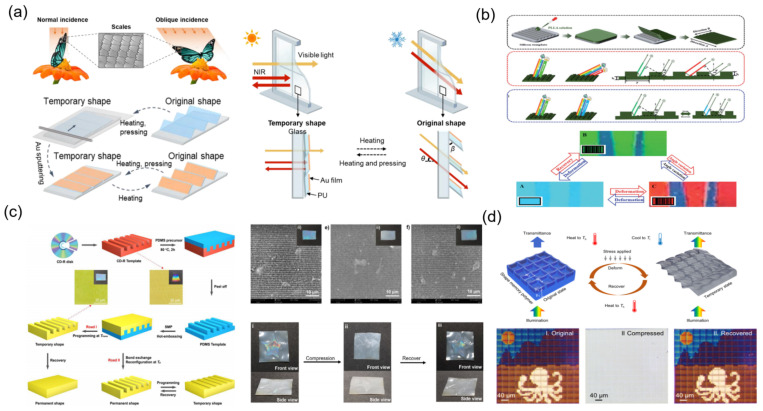
The strategy of thermal driving mode of mechanoresponsive smart windows: (**a**) formation of surface wrinkled structure [58] Copyright 2022, American Chemical Society, (**b**) formation of dynamic network structure [59] Copyright 2022, Royal Society Chemistry (London, UK), (**c**) formation of nanostripe structure [60] Copyright 2022, Elsevier, (**d**) formation of shape memory polymer grids [61], Copyright 2021, Springer Nature.

Building a deformable surface morphology of smart windows using thermo-responsive materials is an effective method to adjust the solar transmission. In Figure 9a, Li et al. used thermo-responsive shape memory polymer and the metal coating, displaying a butterfly wing-like smart window (BSW) [58]. Upon heating, this BSW enable the transition from a temporary flat shape to a predefined tilted configuration. A high solar modulation up to 32.6% and a great luminous transmission up to 64.5% can be achieved. This work provides a novel strategy for thermal driving smart windows. Furthermore, in order to show the color change via thermal driving, Xu et al. designed macroscale and microscale structures through the shape-memory effect based on a poly (*ε*-caprolactone) dynamic network [59].

As illustrated in Figure 9b, upon the thermal stimulus, macro-scale and micro-scale shapes recovered synchronously, and the micro-scale features are modulated for color changes [59]. Inspired by the color change in panther chameleons, Zhao et al. prepared nanostrip patterns on a poly (l-lactic acid) (PLLA) film surface based on the shape memory effect [60]. Various structural colors are displayed, which are decided by the deformation and viewing angle at the macroscale. The structural color with high saturation can be attributed to the periodic patters in nanoscale and the 1D reflective grating. Moreover, the color can be easily tailored by the uniaxial tension. When the stretch ratio is increased, the color turns from red to green and then blue. It can return to red after the shape recovery under heating (Figure 9c). Through two-photon polymerization lithography (TPL), Zhang et al. reported a SMP photoresist with print features at a half pitch of ~300 nm (Figure 9d) [61]. The nanoscale surface structure deformation enables large visual shifts. The colors and printed information are invisible if the nanostructures are flattened. Upon seconds of heating, the initial surface morphology of the nanostructures is recovered due to the shape memory effect, along with its structural color. Thermal driving mode can tune the optical property between the cold and hot states with significant optical performance contrast under appropriate transition temperature while maintaining a high luminous transmission. Further research effort should be made to develop multifunctional mechanoresponsive smart windows. Thermal driving mode windows are a promising approach and further research is needed. However, external thermal control equipment is needed for the practical applications and current research is far from being mature.

### 3.4. Electric Driving Mode

The electric driving mode has the advantage of being precisely controllable and thus has been widely used in the field of electrochromic smart windows [62,63]. The electrically induced dynamic surface/interface to achieve color transformation mainly includes electrically controlled colloidal particle assembly state change, and electrically controlled dielectric elastomer deformation state change.

**Figure 10:**
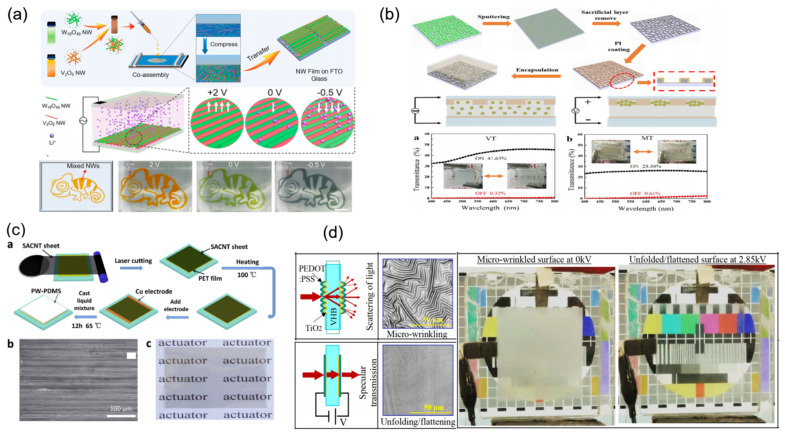
The strategy of electric driving mode mechanoresponsive smart windows: (**a**,**b**) controlling of nanoparticles via the electrophoresis to modulate colors and transmittance [64,65] Copyright 2021, American Chemical Society and 2021, Elsevier, (**c**) carbon nanotube-based materials to adjust transmittance [66] Copyright 2017, Elsevier, (**d**) based on surface wrinkle to modulate transmittance [67] Copyright 2018, American Chemical Society.

As illustrated in Figure 10a, a multicolor electrochromic smart window is exhibited by Wang et al. via co-assembling of W_18_O_49_ and V_2_O_5_ nanowires from the Langmuir–Blodgett technique [64]. With the increase in V_2_O_5_ nanowires, W_18_O_49_ nanowire film turns from optical transparent to orange. A dynamic color change (orange, green, and gray) is also shown in the presence of different electrochemical biases of 2, 0, and −0.5 V. By the manipulating of the co-assembled nanowire layers and the ratios between two nanowires, they can effectively control both the color and transmittance of the smart window film. Different patterns can also be shown with the easy addition of various masks via the same method.

Inspired by the superior optical transmittance of crack pattern and fractal networks, Liu et al. designed a novel category of smart windows based on electrophoresis [65]. The TiO_2_@SiO_2_ particles with core-shell structure are chosen as the electrically responsive medium in this type of smart windows. A strong light scattering of TiO_2_ and a large accumulation of surface charge on SiO_2_ are combined in these particles (Figure 10b). Thus, the uniformity of dispersed solution is improved, and a fast response time to the electric field is achieved. Under the power-off state, the core-shell TiO_2_@SiO_2_ particles are uniformly dispersed, resulting in the strongly suppressed transmission due to light scattering on these particles. Under the power-on state, a weaker dielectric property is obtained due to the thinner polyimide film in the valley. The device transmittance is ultimately improved because the TiO_2_@SiO_2_ particles can be absorbed and squeezed in the valley. Moreover, this intelligent window also exhibits an excellent property in blocking UV and NIR bands of natural light, which could effectively reduce the heating effect. Zhang et al. demonstrated a transparency-switchable actuator from single-layer carbon nanotube (CNT) sheet with super alignment on paraffin–PDMS composite (Figure 10c) [66]. Upon an applied extra voltage, the transmittance of the film changes from 0.7% to 67% (at the wavelength of 550 nm). An obvious bend with a displacement of 8.4 mm is also shown at the same time. The transparent low-cost substrate and adjustable surface roughness via electric field endow this smart window film with easy preparation and convenient modulation. As shown in Figure 10d, Shrestha et al. achieved the transition from transparent to translucent due to the reversible electric field-modulated wrinkling [67]. The optical surface scatterer is electrically adjustable, which includes TiO_2_ thin film with a high refractive index and high modulus. These optical metasurfaces display a decrease in in-line transmittance from 81% to 1.85% under a small radial compression (4–5%). These surfaces’ morphology of microscale wrinkle can be released by reversible expansion due to voltage-induced surface expansion. In addition, the current leakage can be decreased and high-voltage activation can be achieved at a notable low power of 0.83 W/m^2^. In current research, multifunctional electric driving devices have wide applications in self-powered mechanical and solar energies serving as the energy storage devices or actuators. However, there are still problems in the cost-effectiveness, scalability, or reliability. Different strategies and great efforts are still needed to further understand the improved electric driving performance and design of novel devices.

### 3.5. Magnetic Driving Mode

Magnetic field is an effective way to induce directional alignment of magnetic materials, mainly including induced particle directional alignment or assembly, and magnetically induced elastomer directional deformation [68,69]. It has the advantages of non-contact conditions to induce directional deformation of materials, non-invasive, large deformation effect, and precise remote control.

**Figure 11:**
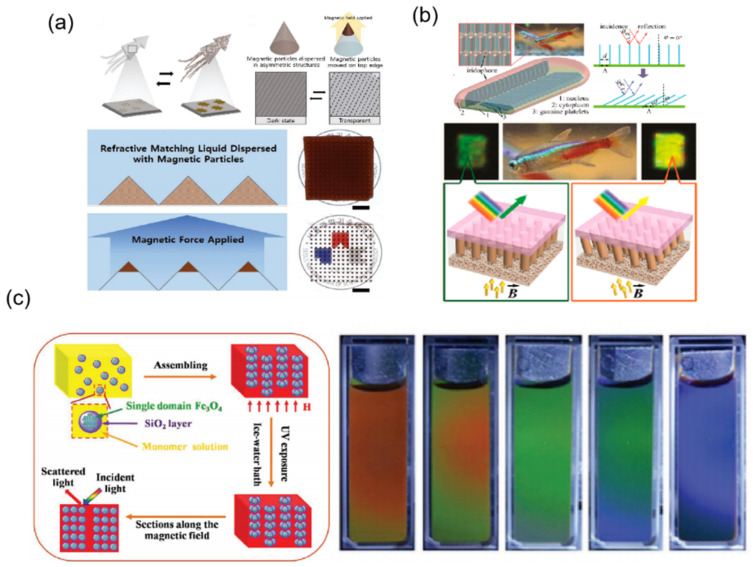
The strategy of magnetic driving mode mechanoresponsive smart windows: (**a**) adjusting the ratio of magnetic nanoparticles to change transmittance [70] Copyright 2019, John Wiley and Sons, (**b**) forming the magnetic nanopillars array to adjust transmittance [71] Copyright 2019, American Chemical Society, (**c**) forming magnetothermal hydrogel to modulate transmittance [72] Copyright 2018, John Wiley and Sons.

Inspired by the transformable skin of squid through the controlling of the light-absorption area, based on the dispersed magnetic nanoparticles in an asymmetric pyramidal array, Yang et al. exhibited a manipulated light-absorption area via magnetic field [70]. The refraction at the interface is avoided due to the refractive index match of dispersion media liquids. The transparency performance is dependent on the ratio of magnetic nanoparticles (Figure 11a). At the same time, inspired by the tropical fish of neon tetra, Luo et al. realized dynamic iridescence via the tune of magnetic field (Figure 11b) [71]. In this strategy, periodic nanopillar arrays serve as a template to induce the assembly of iron oxide nanoparticles while in a liquid environment. Induced by the magnetic template, the periodic local fields anchor the assembled particle columns, enabling the structure to tilt when the applied magnetic field angle is changed. This effect is similar to a microscopic “Venetian blind”, in which dynamic optical properties of structural coloration can be tunable in real time. Tunable reflectance spectra are displayed in the generated prototype with an obvious peak shift from 528 to 720 nm. Especially, the magnetic actuation is reversible and it has a very fast response time of about 0.3 s. In Figure 11c, Wang et al. then demonstrated a new approach for a magnetochromic display based on magnetic photonic crystals hydrogel [72]. The external magnetothermal stimuli can drive the visual color of the hydrogel in the presence of a tunable magnetic field. The magnetic hydrogel’s photonic structures were improved to be aggregates of 1D magnetic chains and were fixed within the thermosensitive hydrogel of PNIPAM. Resulting from the magnetothermal effect of 1D aggregated magnetic chains, the heat was revealed to transfer to the surrounding matrix instantly, leading to a reversible transition of the hydrogel from hydrophilic to hydrophobic. This transition also reduces the distance between particles in the 1D magnetic chains, which subsequently results in an obvious blue shift of diffraction wavelength. Thus, the magnetic hydrogel displays the potential of monitoring magnetic hyperthermia with significant changes in its color appearance. Additionally, this magnetic hydrogel also exhibits excellent stability and repeatability.

Through the modulation of distance or angle between magnetic particles, magnetic driving smart windows can conveniently tune the transmittance reversibility. Through the changes in magnetic field intensity, temperature, or switch status, various colors of the magnetic microspheres can be diffracted. Thus, these magnetic driving intelligent windows display many colors. Due to the response to magnetic fields, these smart windows highlight the potential applications in energy saving buildings, privacy-ensuring windows, etc.

### 3.6. Humidity Driving Mode

Atmospheric humidity is one of richest natural resources; however, it is seldom exploited. The color or transmittance of smart windows can be changed upon the humidity changes. Water adsorption/desorption can be environmentally controlled; thus, there is no need for an external energy supply. While the relative humidity (RH) changes, the scattering efficiency and transmittance of windows also change. An opaque window features material that may become transparent (or vice versa) or change color, depending on the humidity.

**Figure 12:**
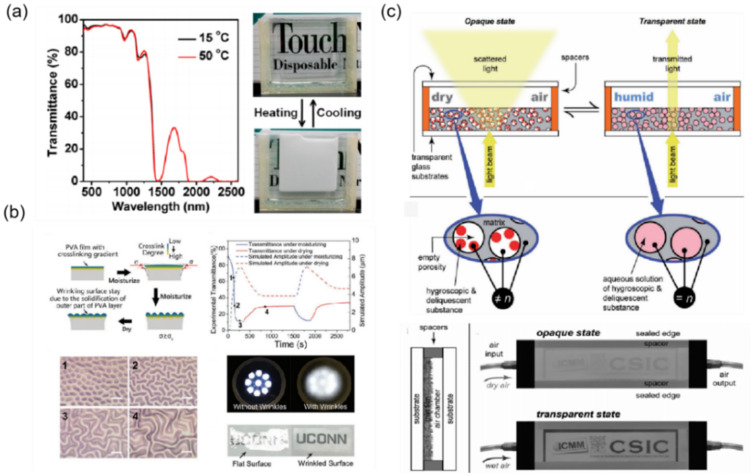
The strategy of humidity driving mode mechanoresponsive smart windows: (**a**) based on thermosensitive hydrogels [73] Copyright 2014, American Chemical Society, (**b**) based on the humidity driving mode wrinkles [74] Copyright 2017, John Wiley and Sons, (**c**) base on humidity sensitive porous structure [75] Copyright 2018, John Wiley and Sons.

As illustrated in Figure 12a, Wang et al. prepared thermosensitive microgel colloids of poly (N-isopropylacrylamide) (PNIPAAm) [73]. Using water–glycerol as the solvent, PNIPAAm microgel colloids present a good compatibility with the thermosensitive hydrogels, which lead to light deterioration of the solar modulation ability. The external stimuli and the structure and mechanical properties of skin affect the wrinkle dynamics (e.g., reversibility and stability) of human skin. Inspired by this tunable response, Zeng et al. realized moisture-responsive wrinkle dynamics [74].

It is important to control the thickness ratios and stiffness of the film and substrate and modulate the crosslink degree or gradient to result in the moisture-dependent modulus and swelling degree changes (Figure 12b) [74]. As illustrated in Figure 12c, Castellón et al. demonstrated a novel optical hybrid thin film which exhibits reversible humidity-driven properties of light transmittance/scattering [75]. This film is made of a dispersive porous structure, with included hygroscopic and deliquescent compounds. These compounds can scavenge water molecules from the humid environment to fill up the pores and the film becomes transparent to the incident light. When the film is exposed to dry air, water molecules are released from the porous structure and the film recovers its initial light scattering properties. Thus, the transparency of thin films can be changed when exposed to wet/dry air with different RH. Via water absorption/desorption, humidity-responsive smart windows are able to change light transmittance, greatly enhancing the energy conservation of buildings. Importantly, no energy is consumed and such windows will find many applications.

A brief comparison among the six driving modes is shown in Table 1. When the pneumatic, electric, and magnetic driving modes are active, additional control equipment is required. The smart materials via optical, thermal, or humidity driving mode are equipment-free, however, suitable for specific regions.

## 4. Applications of Mechanoresponsive Smart Windows

**Figure 13:**
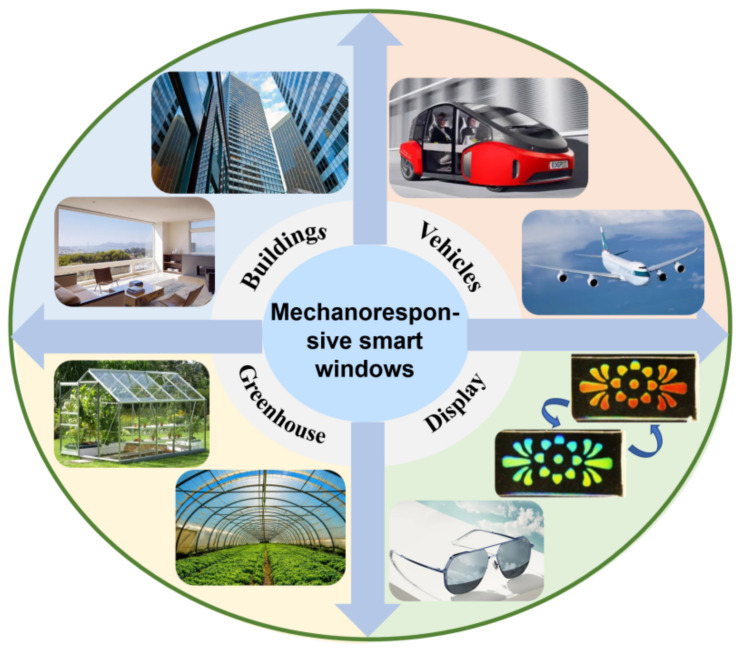
Potential applications of mechanoresponsive smart windows.

Based on the modulation of the surface/interface structure upon mechanical strain, mechanoresponsive smart windows have the ability to change the transparency or color in visible/NIR wavelength. They have unique advantages, i.e., easy construction, low cost, excellent stability, and environmentally friendly. Therefore, mechanoresponsive smart windows show great potential in energy saving, privacy protection, and security, etc. As illustrated in Figure 13, mechanoresponsive smart windows have attracted significant interests in various fields, e.g., buildings, vehicles, greenhouses, and sunglasses.

Mechanoresponsive smart windows that can modulate the light transmittance and heat radiation are crucial for the energy saving in buildings, automobiles, greenhouses, etc. The global smart window market is expected to reach USD 5.0 billion in 2022 and increase to 8.2 billion by 2027, with a compound annual growth rate (CAGR) of 10.4%. More importantly, climate warming is a common problem currently faced by the whole world. The energy saving from the light modulation of smart windows is one of key approaches to solve the global warming crisis. Liu et al. presented three modes for the application of smart windows in greenhouses. In cloudy days, the smart membrane is rolled up, showing a fully transparent state, and providing sufficient solar radiation. In sunny days, the smart membrane displays the relaxed state, in which UV and NIR lights are effectively blocked while visible lights can transmit. In hot days, the high blocking mode is triggered when the smart membrane is stretched and the broadband UV–Vis-NIR radiation could be effectively restrained. Under this mode, the smart membrane can achieve UV protection, lower the indoor temperature, and protect privacy indoors.

Moreover, based on the transparency or color modulation, it can also be used in information transmission, security, and other fields. Li et al. [29] achieved surface wrinkles by the plasma treatment of pre-stretched elastomer, showing the variable color and transparency, which can be applied to multiple security techniques. Zhou et al. developed a film consisting of a brown annular nanoparticle array and transparent elastomer at the top. Due to the large difference in mechanical strength, buckling instability will occur on the surface under compression load, resulting in grating-shaped one-dimensional or two-dimensional wrinkle patterns. By setting different patterns on both sides of the film, specific patterns in different bending directions can be displayed.

Under sunshine, people usually adjust the luminous flux through the size of the pupil. However, when the light intensity exceeds the ability of human eyes, it will cause damage to the human eyes. Especially in the summer, sunglasses are needed to block the sun and reduce eye fatigue or damage. Mechanoresponsive smart windows are environmentally friendly and require no external equipment. The sunglass market of China reached USD 5.2 billion in 2020, with a CAGR of 4.18%. The huge business market is the key driving force for the development of smart windows in sunglasses.

## 5. Conclusions and Perspective

This review summarizes the recent progress of mechanoresponsive smart windows. Based on the optical modulation arising from surface morphology or interface structure under mechanical strain, the mechanoresponsive smart windows have shown unique advantages: simple construction, low cost, high stability, and a fast response time. In this paper, we focus on the structures and mechanisms of each type and also discuss their advantages and disadvantages. Generally, the driving mode to the mechanical strain is the mechanical force, such as tensile, compression, and twisting. Especially, we summarize six unconventional driving modes to the mechanical deformation and provide brief comparison. Generally speaking, compared to other electrochromic, thermochromic smart windows, the mechanoresponsive smart windows exhibit remarkable advantages in cost, construction, or stability. Moreover, the various driving modes to drive the mechanical strain can satisfy needs for different environments. However, challenges remain for the future development of smart window technology to be fully utilized in various fields:

(1) Although a variety of mechanoresponsive materials have shown a wide range of regulation in transmittance, they generally require a large strain for sufficient transmittance change which means more energy consumption. There is a great need for the design and preparation of highly sensitive mechanoresponsive smart windows.
(2) There are several modes to drive the mechanical deformation; however, each mode has its advantages and disadvantages. One of the future trends is to combine several modes in one device. For example, smart windows driven by both mechanical force and light can furtherly reduce the energy consumption. The challenge lies in balancing the cost, construction, and energy saving.
(3) With the higher requirements of buildings, automobiles, and other fields, the demand for multifunctional windows becomes increasingly urgent. Various functions such as energy saving, self-clean, and being colorful, are needed at the same practical circumstance. Thus, the design of low cost but multifunction smart windows is an important trend for the future.

Despite of the considerable challenges we face to facilitate the commercialization of mechanoresponsive smart windows, the excellent stability and simple construction highlight the bright potential of mechanoresponsive smart windows in buildings, greenhouses, vehicles, and displays. We hope that our review will provide a systematic reference for the next generation of mechanochromic smart windows and accelerate their practical development.

## Figures and Tables

**Table 1:** Brief comparison of the six driving modes.

Driving Mode	Actuation	Stimuli	Equipment Complexity Degree	Applicability
Pneumatic	Active	Pneumatic	High	Additional control equipment is required
Optical	Passive	UV/NIR light	Low	Suitable for regions with long sunshine time
Thermal	Passive	Solar radiation	Low	Suitable for tropical regions
Electric	Active	Electric	High	Additional control equipment is required
Magnetic	Active	Magnetic field	Medium	Additional control equipment is required
Humidity	Passive	Water vapor	Low	Suitable for humid regions

## Data Availability

Not applicable.
